# Impacts of plant growth regulators in strawberry plant: A review

**DOI:** 10.1016/j.heliyon.2022.e11959

**Published:** 2022-11-28

**Authors:** Shambhu Katel, Honey Raj Mandal, Sujata Kattel, Shubh Pravat Singh Yadav, Baibhav Sharma Lamshal

**Affiliations:** a G.P. Koirala College of Agriculture and Research Centre (GPCAR), Gothgaun, Morang, Nepal; b Nepal Polytechnic Institute (NPI), Chitwan, Nepal

**Keywords:** *Fragaria × ananassa*, Gibberellin, NAA, TRIA

## Abstract

Strawberry (*Fragaria × ananassa*), the family Rosaceae, is a small fruit that has great importance. It is triggered by a number of physiological, genetic, and biochemical processes. Phytohormones or plant growth regulators are organic substances produced naturally in many plants and responsible for controlling growth and other physiological functions. Therefore, plant growth regulators such as Gibberellin, NAA (auxin) and triacontanol, and chlormequat are essential factors that cause strawberry ripening, maturity indices, and determine the quality of fruits. Moreover, Gibberellin stimulates cell division and breaks dormancy whereas NAA (auxin) stimulates root growth. Similarly, triacontanol plays a special role in plant growth and development. Additionally, chlormequat is effective in controlling the height of the plant. The main objective of this review is to study the effect of various plant growth regulators that have a great potential effect on growth, development, and fruit yield.

## 1 Introduction

**Figure 1:**
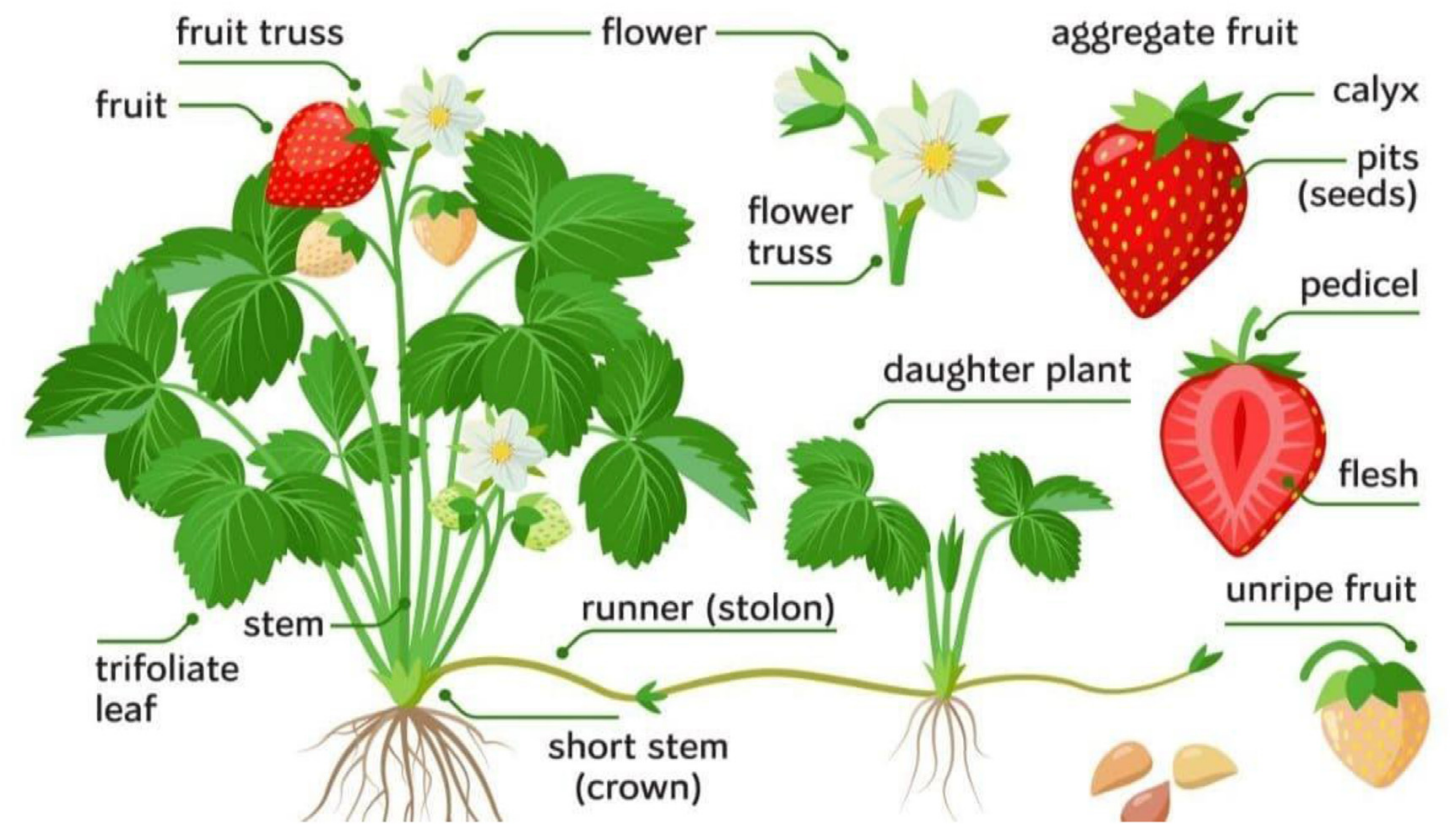
Whole strawberry plant (strawberryplants.org).

Strawberry is soft, luscious, nutritious, tasty, and perishable fruit which are grown in temperate climatic conditions where the plant behaves like a small perennial herb and also grown in a sub-tropical climate whose plant behaves as an annual belonging to the family Rosaceae (Salentijn et al., 2003; Srivastav et al., 2018; Cv et al., 2016). The cultivated strawberry (*Fragaria × ananassa Duch*.) is a monoecious octaploid hybrid of two largely dioecious, octaploid species, *Fragaria chiloensis Duch.* and *Fragaria virginiana Duch* (Cv et al., 2016). Strawberry is a non-climacteric fruit and characterized by a high softening rate, short post-harvest life, and fast decay (Bustamante et al., 2009). Strawberry (*Fragaria ∗ ananassa*) is a short day plant that has antioxidant, anti-inflammatory, anti-neurodegenerative and anti-cancer component called ellagic acid, contain phenolics and flavonoids and also rich in vitamins, minerals like potassium, phosphorus, calcium, and iron (Roussos et al., 2009). It is propagated through the runners and is red in colour due to the presence of anthocyanin, pelarogonidin, 3-monoglucoside, and traces of cyanide (Srivastav et al., 2018). Consumption of strawberries leads to health benefits against cancer, aging, inflammation, and neurological diseases (Cv et al., 2016). *Camarosa, Laguna, Seascape Chandler, Sweet Charlie, Fern, Douglas, Redgauntlet, Talisman, Cambridge Favourite, Domanil, Fanil, Gorella, Goupil, Senga gigana, Senga precosana, Surprise des Hailes* are different cultivars of strawberry (Sharma and Singh, 2009; Paroussi et al., 2002; (Tehranifar and Battey, 1997) Terms, 2017). Strawberry is rich in Vitamin A (60 IU/100 g of edible portion), vitamin C (30–120 mg/100 g of edible portion), fiber, pectin (0.55%) and has a low calorie carbohydrate content and is high in carotenoids, flavonoids, phenols, and glutathione ( Sharma and Negi, 2019; Nautiyal and Shukla, 2015). Strawberry is delicious, nutritious, red fruit and its taste depend upon three compounds i.e. sugar (0.5%), acid (0.90–1.85%), and aromatic compounds (Bj et al., 2020). Strawberry's edible parts are receptacle, petioles, achenes or real fruit, and seed. It has short stems known as crown which produces leaves along the stem axis and flowers (Srivastav et al., 2018) (Figure 1).

Different plant growth regulators perform different function on strawberry. Various PGR like Auxin, gibberellin and cytokinin are used in strawberry in order to increase the fruit size, enhance fruit set, growth, and yields. Among them, auxin are used for enlargement of receptacle, fruit size growth and delay fruit ripening, gibberellin inhibit the fruit ripening, abscisic acid develop a color on fruit and nitric oxide extent the post-harvest life of ripe fruit (Roussos et al., 2009; Marcos et al., 2009). Auxins are also called as key phytohormones, applied in controlling the growth and ripening the fruit which is associated with increase in pectin and reduction of hemicelluloses content (Bustamante et al., 2009).

### 1.1 Influence of gibberellin on strawberry

Plant heights, number of runners, number of flowers, fruit set percentage, number of fruits, fruit size, fruit weight, and fruit quality are all affected by gibberellic acid (Kumra et al., 2018) Gibberellic acid (GA_3_) treatment promoted flowering in non-chilled strawberry plants, shortened the cropping season, and increased vegetative growth and fruit number (Paroussi et al., 2002). It acts as a fruit ripening inhibitor (Marcos et al., 2009). It Increases vegetative development, increases runner formation, lengthens the main stem internode, initiates flower development, promotes stolon formation, petiole length, and leaf area, destroys rosette habit, and slows blossom initiation ( Sharma and Singh, 2009; Guttridge and Thompson, 1964; Tafazoli & Vince-prue, 2015). The effects of a long photoperiod or chilling are also caused by GA_3_ (Guttridge, 1970). Gibberellins are well-known for acting as a long-day hormone in short-day plants. Gibberellin treatment increases vegetative growth but limits flower development (Kender et al., 1971). The GA 3-oxidase enzyme prevents runner and crown branch development, increasing berry output (Hytonen, Elomaa, Moritz and Junttila, 2009). By hydrolyzing protein and releasing tryptophan, GA promotes pollen germination, pollen tube expansion, and auxin biosynthesis. GA_3_ boosts diphenols while inhibiting IAA oxidase activity, resulting in a high auxin level. In the absence of fertilizer, the use of GA resulted in fruit set (Kumar, Saravanan, Bakshi, & Sharma, 2013).

### 1.2 Influence of auxin (NAA) and tricontanol on growth yield and quality

NAA is a synthetic auxin that is most commonly employed in the production of high-quality strawberries in terms of total sugars, ascorbic acid content, and titrable acidity percentage (Bhople et al., 2020). NAA is a synthetic version of auxin that aids in cell elongation, division, vascular tissue differentiation, root initiation, apical dominance, leaf senescence, leaf and fruit abscission, fruit setting ratio, fruit dropping prevention, and flower sex ratio promotion (Mehraj et al., 2015). Naphthalene acetic acid is one of auxin's most important members, and early application of Napthalene acetamide in early stages induces cell division in cambium cells, resulting in the production of xylem tissue in lower internodes, which provides mechanical support to plants while also preventing lodging (Thakur et al., 2017). It has been reported that a medium containing a low concentration of NAA 0.1 mg/l and a relatively high concentration of BA 1.0 mg/l is best for shoot generation, and that a medium containing a high concentration of cytokinin and a low concentration of auxin (a medium with a cytokinin to auxin ratio greater than 1) is best for shoot bud induction. (Lal et al., 2003).

Auxins such as IBA (Indol-3-butyric acid) and NAA (Naphthyl acetic acid) are used to promote rapid and abundant rooting of cuttings from a variety of trees, vines, shrubs, annual and perennial ornamentals (Rademacher, 2015). The effect of NAA on plant growth is greatly reliant on the time and concentration of entry, as well as promoting cellulose production and limiting fruit drop (Suman et al., 2017). GA treatment could only maintain emasculated flower receptacle growth for 6 days, according to Archbold and Dennis (1985), whereas growth of fruit treated with synthetic auxin Naphthalene acetic acid (NAA) could continue for up to 30 days, albeit at a slower rate than pollinated flowers (Roberts and Hooley, 1988). The application of NAA to strawberry fruits enhances fruit size, delays ripening, and boosts anthocyanin accumulation, as well as delaying the flowering time and enhancing fruit output and quality (Csrl, 2016). As stated, using GA_3_ and Napthalene acetic acid alone or in combination enhances plant height, number of crowns, runners, and leaf area. Plants treated with NAA at a concentration of 19.97 mg/l produced berries with the highest total soluble solids, total sugars, and titrable acidity (Kumar and Tripathi, 2009). Because developing leaves are one of the primary sites of auxin biosynthesis, the elongating petiole tissues could directly receive sufficient amounts of auxin from young leaves, resulting in increased petiole length due to rapid cell division and cell enlargement, NAA at 19.97 mg/l and 49.94 mg/l produced significantly longer petioles than the control (Manandhar and Shrestha, 2008). It was reported that using NAA improved the output of berries with greater width and length, as well as weight (Wang et al., 2015). The dormant bud has a lot of auxin activity, while the non-dormant bud has a lot of cytokinin activity, according to researchers. Decapitation and pharmacological studies on dormant buds also demonstrated that reducing auxin levels and administering exogenous cytokinins increases strawberry vegetative shoot regrowth (Li et al., 2021). Auxin plays a vital function in fruit growth and ripening by transcriptionally activating Aux/IAA genes (Liu et al., 2011). The skin hardness and hardiness of the underlying flesh define the firmness of strawberry fruit, and this hardiness is linked to the formation of hard achene growth, resulting in the hardiest fruit in NAA treated plants (Rathod et al., 2021). Tricontanol (TRIA) is a natural plant growth regulator found in epicuticular waxes which is used to increase fruit production. TRIA is a saturated primary alcohol found in epicuticular waxes of a variety of plant species, including *Croton californicus*, Copernica cerifera, and *Jatropha curcas*. It was first discovered in Alfalfa hay (Islam and Mohammad, 2020). Tricontanol is a widely distributed saturated primary alcoholic plant hormone discovered by Chibnallet al.(1933) and has been shown to modulate a variety of physiological and biochemical activities in plants (Zaid et al., 2020). TRIA-mediated improvements in growth yield, photosynthesis, protein synthesis, water and nutrient uptake, nitrogen fixation, enzyme activities, free amino acids, reducing sugars, and soluble protein content have been reported by numbers of researchers (Suman et al., 2017). TRIA affects blossom amount and quality in *Vigna radiata L., Chrysanthemum morifolium* Ramat., *Bougainvillea glabra* Choisy., and *Fragaria ananassa* Duch., which may be connected with TRIA providing an element for bud arrangement, improvement, and better-quality blossoms, as revealed by (Islam and Mohammad, 2020). Photosynthesis has been connected as a key plant reaction to TRIA, greater photosynthesis and higher photosynthate accumulation are connected to increased plant development and dry weight (Altintas, 2011; Emam et al., 2020; Naeem et al., 2012). Plants treated with tricontanol increased root number causing plant to absorb more nutrients from the soil and increased production per plant and similarly the tricontanol treated strawberry had the highest number of fruit, yield per hectare and B:C ratio (KhunteDas et al., 2019a, KhunteDas et al., 2019b). Tricontanol also enhances vital plant physiological processes such as water and mineral nutrient uptake, essential oil yield, secondary metabolites, early bolting, nitrogen assimilation, proline metabolism, and glycine betaine accumulation thereby protecting plants from variety of environment stresses (Zaid et al., 2020). TRIA controls the activation of stress resilience components in farmed plants, which helps the plants to cope with lightning-induced alterations (Islam and Mohammad, 2020; Zaid et al., 2020). TRIA has been shown in several studies to play an important role in regulating a wide range of plant morphological responses, including increasing plant height, biomass, leaf number, and leaf area per plant in most harvests. Foliar application of TRIA up to 1 ppm also resulted in twice the fresh and dry weight of shoot and root of *Solanum lycopersicon* (Roberts and Hooley, 1988). Triacontanol, Activol (GA3, a plant growth regulator), and NAA all increased strawberry vegetative development compared to the control. The largest crown tallness (7.2 cm) was achieved with 100 ppm Activol, although 50 ppm triacontanol treated plants had the most notable leaf number/plant (7.2) and leaf region (49.4 m^2^) (Zaid et al., 2020; Emam et al., 2020; Islam and Mohammad, 2020; Kumra and Reena, 2018; Zaid et al., 2020). Emam et al. (2020) was reported that spraying strawberry cv. with 400 ppm NAA resulted in the greatest natural product width, weight, volume, causticity percent (as citrus extract), and lowest sugar: corrosive ratio in Strawberry cv. Tioga. has increased fruit quality and plant stature, spread, number of leaves per plant, petiole length, leaf region records, days to first blooming and days to fruit bud advancement, fruit yield per plant, best fruit yield per hectare, and days to first blooming and fruit bud advancement in Sweet Charlie with NAA levels of 0, 10, 15, 20, 25, 30, and 35 ppm. A preharvest treatment with NAA 25 ppm has an impact on strawberry cv. Chandler's greater L-ascorbic acid concentration (49.30 mg/100 g mash) after capacity. Moreover, treating strawberries with naphthalene acidic corrosive (NAA) restrains aging and anthocyanin amassing (Ali et al., 2021; Altintas, 2011; Khunte et al., 2020). Whenever strawberries (*Fragaria x ananassa Duch*.) cv. Sweet Charlie plants were treated with triacontanol 5 ppm, the greatest number of natural products (23.31), yield per hectare (27.90 tons), length measurement (1.50), and money-saving advantage proportion (1:3.1) were accounted for (Kumari et al., 2018). Also, Pang and Chen (2020) asserts that TA treatment (50 M) advances fruit improvement by upregulating factors associated with natural product maturing and improvement. The best length measurement proportion of natural product (1.58) was accounted for in research led by (KhunteDas et al., 2019a, KhunteDas et al., 2019b) with the treatment Poultry Manure 2.50 tons + Triacontanol 100 ppm. Besides, a shower got from Moringa leaf separate upgraded strawberry yield, recommending that it very well to be utilized as a foliar splash to accelerate the development of youthful plants (Hind Musa Ibrahim Mohamed, 2021; Islam and Mohammad, 2020). The salicylic corrosive at 2 mM and the Tria at 10 mM medicines showed the most advancement in strawberry vegetative qualities, as well as improved botanical and fruiting characters (Islam and Mohammad, 2020; Khunte et al., 2020; Zaid et al., 2020). At 80 ppm, GA_3_ upgrades vegetative development, sprinter creation (Kumari et al., 2018; Suman et al., 2017b). In contrast with different medicines, research directed by Sachs and Iszak (2015) and Sood et al. (2022) found that consolidating bio-manures and development controllers (i.e., PSB at 6 kg/ha + GA_3_ at 100 ppm) increments normal plant tallness, spread, number of leaves, and leaf region with the briefest opportunity to foster the first sprout. Plants treated with PSB (6 kg/ha) + Triacontanol considerably affected the morphological characteristics of strawberry fruit (5 ppm).

### 1.3 Influence of chlormequat

Plant growth regulators are broadly utilized in fruit crops harvests to advance vegetative development, blossoming, and fruit improvement. Plant development controllers have been found to indirectly affect sprouting by lessening the vegetative turn of events (Islam and Mohammad, 2020; Kumra and Reena, 2018). CCC (Chlormequat), the first plant growth regulator was discovered by professor Tolbert at Michigan state university in the 1950s which is a synthetic PGR antagonist to GAs. The CCC has been shown in studies to efffectively reduce the growth of potato stems, leaves, and runners and thicken the stem of mung bean by controlling vein growth and lodging. Dwarfed plants, thickened stalks, increased chorophyll contents and well developed root systems are results of CCC application ( Liu et al., 2019). Likewise, as indicated by Tiwari et al. (2017) and Zaid et al., 2020a, Zaid et al., 2020b, foliar sprays of (GA3 at 200 ppm) at 30, 60, 90, and 120 days after relocating were deemed the best plant development controllers (PGR's) in terms of vegetative development limits, while (GA3 at 150 ppm) was deemed the best in terms of fruit quality for strawberry development. As per Kumra and Reena (2018), strawberry plants treated with cycocel in September and additionally October yielded before and to some degree more prominent yields in three-year preliminaries. Moreover, contrasted with untreated *Fragaria ananassa, Fragaria ananassa* getting two shower treatments of 10 lM TRIA showed a significant effect on plant tallness and leaf number (Ali et al., 2021; Altintas, 2011; Islam and Mohammad, 2020). As per Deyton et al. (1991), the development of smothering synthetic substances such paclobutrazol aid strawberry creation on plastic. This substance is valuable for limiting the development of sprinters. It created more fruit per plant, in any event, when contrasted with plants on which sprinters had been eliminated manually, and brought about a 22 percent increment in yield. Altintas (2011) and Khunte et al. (2020) observed that under drawn-out day conditions, the most extreme number of days it took to produce the principal blooming strawberry following relocating with 1000 ppm cycocel. Strawberry cv. crown tallness, the number of leaves, and leaf region were completely decreased. Besides, GA_3_ 125 ppm treated strawberry plants had the most extreme plant stature (24.13 cm), while treatment T10 (GA_3_ 75 ppm + CCC 500 ppm) treated strawberry plants had the most elevated length measurement proportion of fruits (2.10). As per the aftereffects of the test directed by Altintas (2011), favorable to Calcium (Ca) has a restraining impact on stem stretching with no adverse consequence on absolute and early yields and fruit quality for quite a long time; furthermore, with 100, and 300 mg l^-1^supportive of Calcium applications presenting a benefit over control plants, they would be adequate to control stem prolongation.

Crops grown with chlormequat chloride have shorter internodes but thicker, darker leaves and the chemical control of plant growth to reduce size through the use of PGR is a common practice to make plant more compact and commercially acceptable (Kumra et al., 2018). High NPK increased both leaf area and adjusted leaf area with CCC treated plants benefitting more than PP333 treated plants (McArthur and Eaton, 1988). In Europe cereal production chlormequat chloride was the first PGR to be used on large scale as antilodging agent. Similarly, chlormequat chloride applied in winter wheat at early stage of tillering increases the number of fertile tillers also reducing length of stem (Rademacher, 2015). Strawberry vegetative growth has been found to be aided by GA_3_ and the use of cycocel increased strawberry yield and quality. The use of cycocel at 500 ppm increased the number of flowers, fruit per plant and yield followed by NAA at 30 ppm (Kumar et al., 2012).

### 1.4 Effect of plant growth regulators on quality of strawberry

The physical and chemical features of the strawberry fruit are modified by the use of growth regulators. The use of plant growth regulators improves the quality of strawberry fruits, according to several studies. All doses of GA_3_ improves strawberry vegetative development, but cycocel at 500 ppm, followed by NAA at 30 ppm, is the best in terms of strawberry output and quality (Bakshi, 2018; Sood et al., 2022). In strawberries, skin hardness is connected to the production of hard achenes, and auxin is known to control this process, resulting in the hardiest fruit in NAA-treated plants. The use of NAA in strawberry plants raises the TSS level by increasing the concentration of volatile compounds as well as the hydrolysis of starchy compounds (Rathod et al., 2021; Saima et al., 2014; Thakur et al., 2017). Likewise, Strawberry is also quite susceptible to salt, and it has been noted that the quality of strawberry fruit has decreased. The ratio of TSS to TA has a significant impact on strawberry flavor. This ratio tended to grow when 30 mg L^−1^ PP333 is applied in both saline and non-saline environments (Jamalian et al., 2008). (Rathod et al., 2021) discovered that foliar spraying of plant growth regulators did not affect the firmness and acidity of strawberry fruits. The plants treated with 125 mg L^−1^NAA, on the other hand, generate the hardiest fruit. With a foliar spray of 125 mg L^−1^ NAA, strawberry fruits had the highest TSS and sugar content. Similarly, Nautiyal & Shukla (2015) claimed that the use of nitrogen-fixing bacteria and GA3 with a lower nitrogen dosage may have a regulatory effect on the absorption and translocation of several metabolites, the most significant of which is carbohydrates, which influences the quality of fruits. Furthermore, SA_3_ and SA_4_ treatments increased certain fruit-quality parameters like TSS, AA, fruit color, and strawberry production (Babalar and Asghari, 2007; Karlidag et al., 2011). SA therapy improves overall quality, and SA at 2 mmol L^−1^ is the most effective without causing any side effects, although SA at 4 mmol L^−1^ causes some fruit damage.

## 2 Conclusion

Plant growth regulators are the tools in flowering, fruiting, and ripening. The use of PGRs is increasing day by day mainly in many agricultural fruit crops. Therefore, numbers of synthetic chemicals are used for the regulations of growth and development of cultivated plants. Moreover, these growth regulators can be utilized for sustainable and ecologically sound fruit production. In addition, promote the less use of chemical fertilizers to a great extent. The review focuses on the influence of PGRs on growth, yield, and fruit quality of fruit crops.

## Declarations

### Author contribution statement

All authors listed have significantly contributed to the development and the writing of this article.

### Funding statement

This research did not receive any specific grant from funding agencies in the public, commercial, or not-for-profit sectors.

### Data availability statement

No data was used for the research described in the article.

### Declaration of interest’s statement

The authors declare no conflict of interest.

### Additional information

No additional information is available for this paper.
